# HIV Nef and Antiretroviral Therapy Have an Inhibitory Effect on Autophagy in Human Astrocytes that May Contribute to HIV-Associated Neurocognitive Disorders

**DOI:** 10.3390/cells9061426

**Published:** 2020-06-09

**Authors:** Laura Cheney, Hillary Guzik, Frank P. Macaluso, Fernando Macian, Ana Maria Cuervo, Joan W. Berman

**Affiliations:** 1 Department of Medicine, Division of Infectious Diseases, Montefiore Medical Center and Albert Einstein College of Medicine, 1300 Morris Park Ave, Bronx, NY 10461, USA; 2 Analytical Imaging Facility, Montefiore Medical Center and Albert Einstein College of Medicine, 1300 Morris Park Ave, Bronx, NY 10461, USA; hillary.guzik@einsteinmed.org (H.G.); frank.macaluso@einsteinmed.org (F.P.M.); 3 Department of Anatomy and Structural Biology, Montefiore Medical Center and Albert Einstein College of Medicine, 1300 Morris Park Ave, Bronx, NY 10461, USA; 4 Department of Pathology, Montefiore Medical Center and Albert Einstein College of Medicine, 1300 Morris Park Ave, Bronx, NY 10461, USA; fernando.macian@einsteinmed.org (F.M.); joan.berman@einsteinmed.org (J.W.B.); 5 Department of Developmental and Molecular Biology, Montefiore Medical Center and Albert Einstein College of Medicine, 1300 Morris Park Ave, Bronx, NY 10461, USA; ana-maria.cuervo@einsteinmed.org

**Keywords:** HIV-associated neurocognitive disorders, Nef, astrocytes, antiretroviral therapy, autophagy, LC3, p62/SQTM1

## Abstract

A significant number of people living with HIV (PLWH) develop HIV-associated neurocognitive disorders (HAND) despite highly effective antiretroviral therapy (ART). Dysregulated macroautophagy (autophagy) is implicated in HAND pathogenesis. The viral protein Nef, expressed even with suppressive ART, and certain antiretrovirals affect autophagy in non-CNS cells. Astrocytes, vital for CNS microenvironment homeostasis and neuronal health, require autophagy for their own homeostasis. We hypothesized that extracellular Nef and/or ART impact astrocyte autophagy, thus contributing to HAND. We studied in-bulk and selective autophagic flux in primary human astrocytes treated with extracellular Nef and/or a combination of tenofovir+emtricitabine+raltegravir (ART) using Western blotting, a tandem fluorescent LC3 reporter, and transmission electron microscopy/morphometry. We show that after 24 h treatment, Nef and ART decrease autophagosomes through different mechanisms. While Nef accelerates autophagosome degradation without inducing autophagosome formation, ART inhibits autophagosome formation. Combination Nef+ART further depletes autophagosomes by inducing both abnormalities. Additionally, extracellular Nef and/or ART inhibit lysosomal degradation of p62, indicating Nef and/or ART affect in-bulk and selective autophagy differently. Dysregulation of both autophagic processes is maintained after 7 days of Nef and/or ART treatment. Persistent autophagy dysregulation due to chronic Nef and/or ART exposure may ultimately result in astrocyte and neuronal dysfunction, contributing to HAND.

## 1. Introduction

Antiretroviral therapy (ART) has dramatically improved the lifespan of people living with Human Immunodeficiency Virus (PLWH). The population of PLWH, however, is developing a variety of age-related comorbidities at an accelerated rate relative to those without HIV infection. One such comorbidity is HIV-associated neurocognitive disorders (HAND). HAND is a spectrum of behavioral, psychomotor, and cognitive abnormalities, ranging from subclinical cognitive dysfunction to frank dementia. Although ART has changed the landscape of cognitive dysfunction from a predominance of dementia to mainly milder forms of deficits, HAND still affects 15–55% of people despite virologic control with ART [1,2,3,4,5,6]. HAND is highly burdensome. Compared to PLWH without HAND, PLWH with cognitive deficits have lower quality of life, with difficulties in interpersonal functioning, employment, and medication adherence [7,8,9,10]. HAND is an independent risk factor for mortality even in the current ART era [11,12,13]. Understanding HAND pathogenesis, which is incompletely characterized, is key to developing specific treatments to mitigate this serious comorbid condition of HIV infection.

Astrocytes are the most abundant cell type in the brain. They serve numerous functions in CNS physiology, globally for structure and blood–brain barrier integrity, nervous system repair, and immunity, and also in the neuronal microenvironment by supplying energy substrates, recycling neurotransmitters, protecting against reactive oxygen species (ROS), and balancing pH [14]. Astrocytes are believed to be infected with HIV at a low level [15,16,17,18,19,20,21], serving as one of the CNS reservoirs for HIV, among macrophages and microglia. Reservoirs are not eliminated by ART.

The inability to eradicate viral reservoirs results in ongoing cell exposure to toxic viral proteins, including HIV Nef. Nef is a small protein without enzymatic activity that impacts many cell processes by interacting with a multitude of cell proteins. It is expressed extracellularly by infected cells even in the presence of suppressive ART, including by astrocytes, and extracellular Nef, by action at the cell surface or by entering into neighboring cells, causes altered functioning, cell injury, and death [22,23,24,25,26,27,28,29,30,31,32]. Intracellular and extracellular Nef are believed to contribute to the development of HAND. For example, its detection in astrocytes correlates with dementia in people who had Acquired Immunodeficiency Syndrome (AIDS) [17]. Rats transplanted with Nef-expressing astrocytes experience neuronal loss, and spatial and object recognition deficits [33]. Exogenous Nef is toxic to neurons in culture [34], and Nef-expressing neurons undergo axonal and neurite degeneration [28]. Human brain microvascular endothelial cells undergo apoptosis when exposed to extracellular Nef [35].

That HIV establishes reservoirs also necessitates lifelong treatment with antiretroviral compounds, which themselves can disrupt CNS cell homeostasis. Tenofovir and emtricitabine, nucleoside reverse transcriptase inhibitors that comprise the backbone of most current ART regimens, decrease human astrocyte proliferation and increase expression of cell cycle inhibitor p21, features consistent with cell senescence [36]. Lopinavir, a protease inhibitor, disrupts glutamatergic signaling in and uptake by human astrocytes [37]. Emtricitabine and lopinavir impair astrocyte cell membrane integrity [38]. Off-target effects of ART on astrocytes possibly contribute to HAND pathogenesis, yet the neurotoxicity of ART and that of Nef are not completely defined.

One very important function necessary for astrocyte homeostasis that we hypothesize could be impacted by Nef and ART, and therefore contribute to HAND, is macroautophagy, hereafter called autophagy. Autophagy is a highly regulated process by which organelles and macromolecules are degraded in lysosomes as part of cellular quality control [39]. Autophagy is initiated by the formation of a double-membrane vesicle, an autophagosome (APG), that sequesters organelles and proteins (cargo) targeted for lysosomal degradation. Cargo can be included in the APG non-selectively, termed “in-bulk” autophagy, in that the APG engulfs cytosolic components that are nearby [40]. It can also be selective, whereby autophagy adapters and/or receptors bring selected cargo to the forming APG for lysosomal degradation of specific cytosol material [40]. Once fully formed, the APG fuses with a lysosome and the cargo inside is rapidly degraded by lysosomal proteases. The resulting catabolites are recycled out of the lysosome as building blocks for cellular metabolism. The rate at which this process progresses from start to finish is termed autophagic flux [40]. Flux occurs constitutively, at a basal level, for turnover of intracellular proteins and organelles, and therefore maintains cell homeostasis. Autophagy is also induced by a variety of stimuli that perturb homeostasis, such as starvation, oxidative stress, hypoxia, proteotoxicity, lipid insults and pathogens, among others. Balance of APG formation with APG degradation is vital for maintenance of the cytosolic environment and proper cargo disposal. Disruptions to autophagic activity, specifically in astrocytes, have been linked to the pathogenesis of several neurodegenerative conditions, including Amyotrophic Lateral Sclerosis (ALS), and Huntington’s and Parkinson’s diseases [41,42,43,44,45,46].

Nef as well as certain antiretroviral drugs impact autophagy [47,48,49,50,51,52,53]. A few studies have examined the relationship between autophagy and HIV-induced and/or ART-induced neuropathology [54,55,56,57,58,59,60,61], but very few have examined how dysregulated autophagy in astrocytes by HIV proteins and/or ART may contribute to HAND pathogenesis [28,62,63,64,65]. Given the significant prevalence of cognitive dysfunction in PLWH who take ART, and the importance of autophagy to astrocyte homeostasis, we propose that extracellular Nef and ART impact autophagy in astrocytes, contributing to neurodegeneration and HAND. Therefore, we studied the effects of Nef, a common ART regimen, and Nef and ART together on autophagy in primary human astrocytes. Our results show that Nef and/or ART induce imbalance in autophagy. Specifically, Nef accelerates APG degradation without inducing APG formation. ART inhibits APG biogenesis without decreasing degradation, and Nef+ART induce both changes in autophagy. We also show that Nef and/or ART inhibit lysosomal degradation of p62/SQTM1, which indicates that they also affect selective autophagy. These findings demonstrate a novel mechanism by which HIV, and its treatment, may contribute to neurodegeneration and cognitive dysfunction in PLWH.

## 2. Materials and Methods

### 2.1. Cells and Treatments

Primary human astrocytes were isolated as previously described [66]. Several different astrocyte lineages were used for experiments. Cells were maintained in Dulbecco’s Modified Eagle Medium (DMEM), supplemented with 10% fetal bovine serum (FBS) and 5% penicillin/streptomycin at 37 °C with 5% CO_2_ atmosphere. Confluent astrocytes were treated with 100 ng/mL HIV Nef (HIV-1 ARMA029 Nef Recombinant Protein, NIH AIDS Reagent Program), and/or with 5 ng/mL tenofovir + 109 ng/mL emtricitabine + 14.5 ng/mL raltegravir (ART) for the indicated durations. The antiretroviral concentrations are consistent with average drug levels achieved in human cerebral spinal fluid (CSF) [67]. Antiretroviral compounds were obtained through the NIH AIDS Reagent Program, Division of AIDS, NIAID, NIH as follows: tenofovir (Cat# 10199); emtricitabine (Cat# 10071); and raltegravir (Cat# 11680). Nef and all three antiretrovirals were resuspended in water, and aliquots of stock concentrations were stored at −80 and −20 °C, respectively. Media were aspirated and replaced with fresh DMEM prior to the start of all treatments. For treatments lasting 7 days, media were changed every 4 days, with treatments added daily.

### 2.2. Western Blotting

Astrocytes were treated with Nef and/or ART for 24 h or daily for 7 days. Untreated cells were used as control. 30–70 µM chloroquine (CQ) were added to some of the samples for the last 2 or 4 h of treatment to inhibit lysosome degradation and assess autophagic flux [68,69]. At treatment completion, cells were washed with ice-cold PBS, and then lysed with Radioimmunoprecipitation Assay (RIPA) buffer containing 1× Halt Protease Inhibitor and Phosphatase Inhibitor Cocktail (Thermo Fisher Scientific). Protein concentration was determined by the Bradford method with Protein Assay Reagent Concentrate (Bio-Rad). Equal amounts of protein were resolved by sodium dodecyl sulfate poly-acrylamide gel electrophoresis (SDS-PAGE) under reducing conditions, followed by transfer overnight at 4 °C to activated poly-vinylidene difluoride (PVDF) membranes. Revert Total Protein Stain (Li-Cor) was used to determine total protein optical density (O.D.) following transfer, using the very sensitive Odyssey Fc System (Li-Cor) for visualization, and Image Studio v. 5.2 software (Li-Cor) for O.D. measurement. Membranes were blocked in 5% non-fat dry milk in 1× TBS plus 0.1% Tween-20 prior to overnight incubation at 4 °C with rabbit anti-LC3 (Cell Signal #2775) or with rabbit anti-p62 (Enzo BML-PW9860) at dilutions of 1:1000. HRP-conjugated goat anti-rabbit antibody (Cell Signal #7074) was used as the secondary antibody for both LC3-II and p62 blots, also at 1:1000 dilution. Blots were developed using 1:1 Super Signal West Femto Chemiluminescent Substrate and Luminol/Enhancer (Thermo Fisher Scientific), and visualized and analyzed the same as for total protein. Primary human cells are inherently variable. To address this variability, LC3-II and p62 O.D. were normalized with two approaches. First, LC3-II and p62 were normalized to total protein O.D. We normalized to total protein because it is an accurate representation of global protein expression within the cell, compared to protein products of housekeeping genes such as GAPDH or actin, which are degraded by lysosomal and non-lysosomal mechanisms, and could thereby skew the normalization of LC3-II or p62 [68]. Second, we normalized LC3-II and p62 to a select 25 kDa band on the total protein stain. This band did not vary by more than 10% across all lanes on 6 different blots, suggesting that the proteins in this region were not significantly impacted by our treatments or changes in autophagy. This band was then used to normalize LC3-II and p62 for all blots. 150 LC3-II values normalized to total protein were then randomly selected for statistical comparison to the corresponding LC3-II values normalized to the 25 kDa band. To determine changes in autophagic flux, LC3-II was analyzed four ways, as suggested in [68,69]. First, the LC3-II level (steady state) was determined as the amount of normalized LC3-II in cells not treated with CQ. Next, APG biogenesis was determined by subtracting normalized LC3-II in cells treated with CQ for 2 h from LC3-II in cells treated with CQ for 4 h. Third, degradation rate (flux) was determined by dividing the normalized LC3-II in cells with CQ for 4 h by the LC3-II in cells without CQ. Fourth, degradation amount (net flux) was determined by subtracting LC3-II in cells without CQ from LC3-II in cells treated with CQ for 4 h. Flux and net flux were determined only with 4 h CQ inhibition. The 2 h CQ inhibition was only used for calculation of APG biogenesis. Changes in overall cellular content (steady state) and rates of p62 degradation in lysosomes (flux, and net flux) were determined in a similar fashion. The effect of any treatment (Nef, ART, or Nef+ART) was calculated as a fold change relative to untreated control cells.

### 2.3. Fluorescence Microscopy

Astrocytes were transduced in 6-well plates at a density of 2 × 10^5^ with a 1:4 dilution in cell medium of lentivirus carrying the tandem mCherry–GFP–LC3 construct [70]. Transduced cells were allowed to express the dual fluorescent LC3 protein for 23 days prior to transfer to 12 mm poly-d-lysine coated glass coverslips (Corning). Once adhered, cells were treated with Nef and/or ART for 24 h. One additional set of cells was treated for 16 h with 2.5 µM rapamycin, a known autophagy inducer, and another set with 70 µM CQ for 4 h, an autophagy inhibitor. Although manipulation of autophagy with rapamycin or CQ is not needed to evaluate autophagy when using the tandem fluorescent reporter, we included these treatments as controls to show that astrocytes were appropriately responsive to pharmacologic manipulation. Transduced untreated, and untransduced untreated cells also served as controls. After treatments, cells were fixed in 4% Paraformaldehyde/PBS for 20 min, washed twice with cold PBS, and then mounted onto glass slides with Prolong Diamond Mountant with 4′,6-diamidino-2-phenylindole (DAPI) (Invitrogen). Three independent experiments were performed. In total, 6080 non-saturated images of individual cells for each treatment condition and control from each of the three experiments were taken using the 63x oil objective 1.4 na, on the Zeiss AxioObserver microscope (Zeiss). Images were analyzed using Volocity Quantitation software (Quorum Technologies). Briefly, the average number of red and green puncta per cell was quantified in the cytosol using the measure object algorithm after establishing appropriate fluorescent thresholds for puncta detection for each individual experiment. Quantification of the total number of red puncta was used to determine the total number of autophagic vesicles (AV = APG + AL). Puncta positive for both fluorophores correspond to APG, whereas those positive only for mCherry correspond to AL. Images were captured and analyzed in a blinded fashion. Puncta were counted in untransduced cells using the same threshold algorithm determined for each experiment. There were negligible numbers of puncta in untransduced cells.

### 2.4. Transmission Electron Microscopy

Cells were treated with Nef and/or ART for 24 h. Untreated cells were used as control. After treatment, cell monolayers were fixed with 2.5% glutaraldehyde in 0.1 M sodium cacodylate buffer, post-fixed with 1% osmium tetroxide followed by 2% uranyl acetate, then dehydrated through a graded series of ethanol. Cells were lifted with propylene oxide and embedded as a loose pellet in LX112 resin (LADD Research Industries). Ultrathin sections were cut on a Leica UC7 ultramicrotome, and stained with uranyl acetate followed by lead citrate. Images were captured on a JEOL 1400 Plus transmission electron microscope at 120 kv. Two independent experiments were performed, and twelve to eighteen images per treatment condition were obtained. A priori defined criteria for organelle scoring [71,72,73] were applied to each image to determine the number of APG and total lysosomes per µm^2^ cytosol, which was determined using Adobe Photoshop (Adobe), discounting the area of the nucleus when present in an image. Images were captured and analyzed in a blinded fashion.

### 2.5. qRT-PCR

Cells were treated for 8 or 24 h, or daily for 7 days with Nef and/or ART. Untreated cells were used as control. At treatment completion, cells were washed in ice-cold PBS, and total RNA isolated with Trizol reagent, according to the manufacturer’s protocol (Thermo Fisher Scientific), including the chloroform extraction step. RNA was eluted in RNase free water (Ambion) and stored at −80 °C. RNA was quantified using a NanoDrop 2000 Spectrophotometer (Thermo Fisher Scientific). 2 µg RNA were reverse transcribed into cDNA using SuperScript Vilo Master Mix (Invitrogen), according to the manufacturer’s protocol, and cDNA was stored at −20 °C if not used immediately for qRT-PCR. Taqman Gene Expression Assays for human 18S and p62 (Applied Biosystems) were performed using Taqman Gene Expression Master Mix on a StepOne Plus Real-Time PCR system (Applied Biosystems). PCR cycling conditions were as recommended for Taqman Assays. The relative quantity of p62 mRNA in treated cells was calculated using the 2^−ΔΔCt^ method relative to the same mRNA in control cells, with 18S serving as the reference gene for treated and control cells.

### 2.6. Statistical Analysis

Data were analyzed using GraphPad Prism software v. 8.4.2 (GraphPad). Data were tested for normality using the D’Agostino and Pearson normality test. Paired T-tests were used to compare LC3-II or p62 values normalized to total protein to the corresponding LC3-II or p62 numbers normalized to the 25 kDa band. For this specific test, a value of *p* < 0.1 was considered significant. When normally distributed, experimental treatments were compared to controls by T-test, and the Wilcoxon Matched-Pairs Signed Rank test when not normally distributed. For fold-change analyses, One-Sample T-tests were used for normally distributed data, and the Wilcoxon Signed Rank test was used for data not normally distributed. Comparisons were made between treatment and control, which was set to a theoretical mean of 1. Values of *p* < 0.05 were considered significant.

## 3. Results

### 3.1. Nef and/or ART Imbalance Autophagy

Nef is produced by infected cells, and present in the extracellular space, even when viremia is suppressed by ART [22,23,24,25,26,27,28]. Extracellular Nef can alter cell function [23,26,28,29,30,31,32]. Autophagy alterations are increasingly recognized as a contributing factor of HAND, yet little is understood regarding the impact of extracellular Nef and/or ART on autophagy in astrocytes, an essential cellular process of which dysregulation is linked to neurodegeneration. To address this, we performed Western blotting for the well-established APG marker, LC3-II. LC3 undergoes cleavage and conjugation to phosphatidylethanolamine to form LC3-II, which is required for autophagosome formation. LC3-II associates with both sides of the APG membrane, and the fraction of LC3-II on the inner APG membrane is degraded when the APG fuses with the lysosome. To determine APG biogenesis, and also rate and amount of APG degradation, we measured changes in LC3-II levels between cells treated or not with chloroquine (CQ) to inhibit lysosome degradation, as described in Methods and elsewhere [68,69]. We show data resulting from normalization to total protein. There was no significant difference between LC3-II normalized to total protein relative to LC3-II normalized to the 25 kDa band (*p* = 0.15; Figure S1).

**Figure 1:**
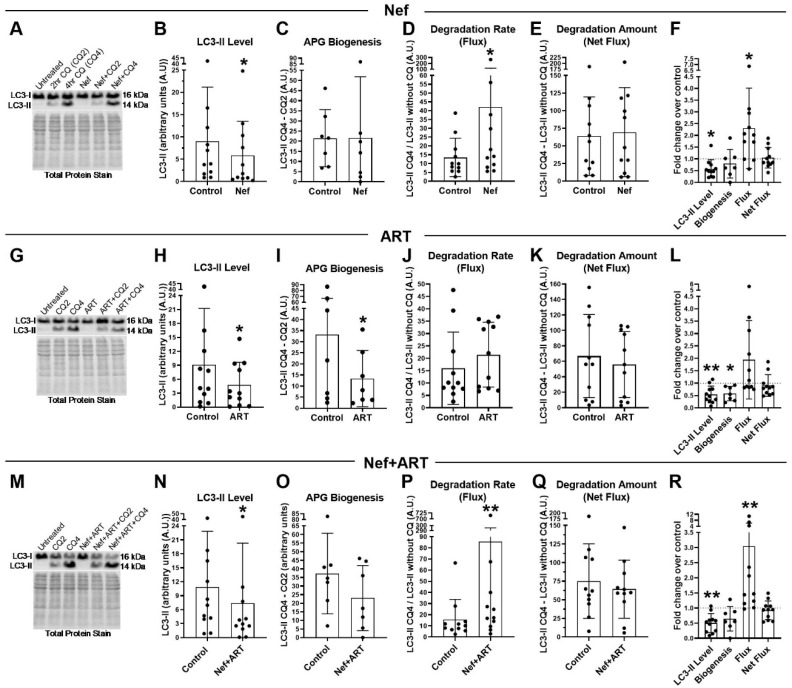
LC3-II steady-state level, autophagosome (APG) biogenesis and APG degradation after treatment with Nef and/or antiretroviral therapy (ART) for 24 h. Primary human astrocytes were treated daily for 7 days with extracellular Nef and/or ART, without and with chloroquine (CQ) for the last 2 or 4 h, and then LC3 Western blotting was performed. Steady-state level, biogenesis, flux and net flux for LC3-II were calculated as described in Methods. (**A**,**G**,**M**) Representative LC3-II Western blots after A. Nef, G. ART, or M. Nef+ART treatment. B–E, H–K, and N–Q show individual experiments (dots) and the means of the experiments (bars). (**B**,**H**,**N**) LC3-II steady-state level in control and B. Nef, H. ART, or N. Nef+ART treated astrocytes. (**C**,**I**,**O**) APG biogenesis in control and C. Nef, I. ART, or O. Nef+ART treated cells. (**D**,**J**,**P**) Rate of APG degradation (Flux) in control and D. Nef, J. ART, P. Nef+ART treated astrocytes. (**E**,**K**,**Q**) Amount of APG degradation (net flux) in control and E. Nef, K. ART, or Q. Nef+ART treated cells. (**F**,**L**,**R**) show the fold changes of the same autophagy parameters after treatment with F. Nef, L. ART, and R. Nef+ART over controls of individual experiments (dots) and the means of the fold changes of the experiments (bars). Controls were set to 1, represented by the dashed lines. Error bars depict SD. *n* = 7–11; *, *p* < 0.05; **, *p* < 0.005 by the Wilcoxon Matched-Pairs Signed Rank test or the Wilcoxon Signed Rank test.

**Figure 2:**
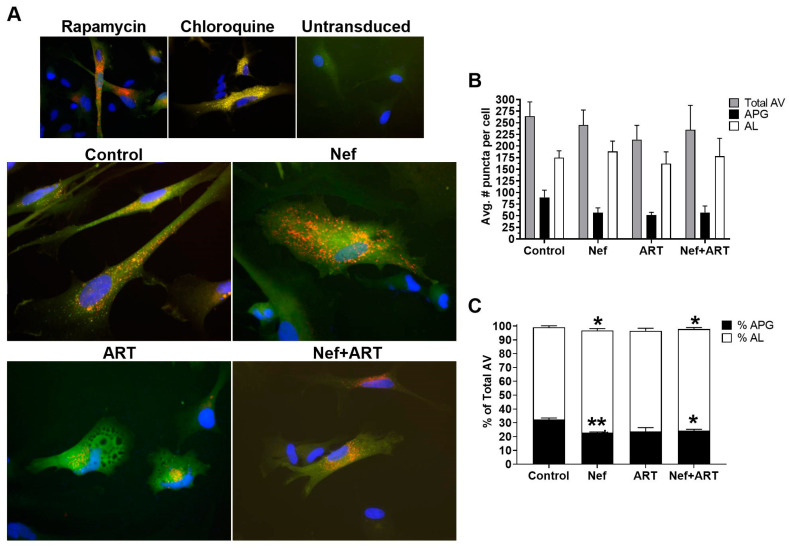
Autophagic vesicles (AV) after treatment with Nef and/or ART. Primary human astrocytes were transduced with a lentivirus expressing the mCherry–GFP–LC3 autophagy reporter, and treated with rapamycin for 16 h, chloroquine for 4 h, or with extracellular Nef and/or ART for 24 h. Red and green puncta were analyzed as described in Methods. (**A**) Representative images from transduced and treated cells, and untransduced cells. (**B**) The mean number of puncta per cell in control, Nef, ART, and Nef+ART treated cells. (**C**) Mean percent APG and AL of total AV in control, Nef, ART, and Nef+ART treated cells. Error bars represent SEM. *n* = 3 independent experiments, with a total of 189–229 cells per condition analyzed; *, *p* < 0.05; **, *p* < 0.005 by the Wilcoxon Matched-Pairs Signed Rank test.

Treatment with Nef for 24 h without CQ resulted in a significant 35% decrease in mean LC3-II steady-state level compared to control (Figure 1A,B; *p* < 0.05), with a mean 0.42-fold reduction (Figure 1F; *p* < 0.05). This decrease could be due to decreased APG biogenesis and/or increased APG degradation. Western blot analyses comparing LC3-II at two different points after addition of CQ demonstrated no differences in APG biogenesis, measured as the difference in LC3-II levels between 4 h and 2 h of CQ treatment (Figure 1A,C). Instead, we observed that the decrease in LC3-II was due to accelerated APG clearance (flux) (Figure 1A,D,E) as Nef significantly accelerated autophagic flux (Figure 1A,D; *p* < 0.05), 2.3-fold above control (Figure 2F; *p* < 0.05). Despite this increase in autophagic flux, we observed that the total amount of APG degraded (net flux) after Nef treatment was not different from control (mean 69.5 compared to control mean of 63.82, Figure 1A,D, or 1.1-fold that of control, Figure 1F). This further supports that the reduction in LC3-II was due to accelerated degradation without changes in APG formation. These data indicate that Nef increased efficiency of APG degradation but not the overall amount of degradation by autophagy since it was not associated with an accompanying induction of APG formation.

We performed similar studies with ART. Treatment with ART for 24 h also resulted in a significant 47% decrease in LC3-II steady-state level relative to control (Figure 1G,H; *p* < 0.05; or 0.46-fold that of control, Figure 1L; *p* < 0.005). This decrease, unlike for Nef, which caused accelerated degradation, was due to a significant 60% decrease in APG biogenesis (Figure 1G,I; *p* < 0.05; or 0.36-fold less than control; *p* < 0.05). With biogenesis significantly reduced, we anticipated a decreased amount of APG degradation (net flux). That is, if there are substantially fewer APG formed, this will lead to less APG maturing into AL. In agreement with decreased biogenesis, we found a trend towards reduced net flux after ART treatment relative to control (mean 55.9 compared to control mean of 66.9, Figure 1G,K; or 10% less than control, Figure 1L), thus suggesting decreased biogenesis after ART treatment. Similar to Nef, we observed a trend towards accelerated LC3-II degradation rate (flux) that was 134% higher in ART-treated than in control cells (Figure 1J; 1.9-fold over control, Figure 1L). This average was driven upwards by a group that demonstrated a striking increase in the fold change in flux relative to control (3.2-fold increase, *n* = 5, Figure 1L). This is in contrast to another group in which the fold change in flux was not changed relative to control (mean 0.88-fold, *n* = 6, Figure 1L). These data demonstrate that ART consistently inhibited APG biogenesis, but there is a variable response to the drugs with regards to APG clearance. It is possible that individuals may have different capacity for degradation in presence of antiretroviral drugs.

Concomitant treatment with Nef and ART for 24 h resulted in effects on biogenesis and degradation similar to the sum of the affects shown after individual treatments with Nef or ART. Specifically, Nef+ART treatment caused a significant 32% decrease in LC3-II steady-state level relative to control (Figure 1M,N; *p* < 0.05), or mean 0.5-fold less than control (Figure 1R; *p* < 0.005). This decrease was a consequence of the 38% reduction in APG biogenesis (Figure 1M,O), and a 3-fold acceleration of APG degradation (flux) over control (Figure 1P,R; *p* < 0.005). As expected from the reduced biogenesis and accelerated clearance, the net flux was 28% decreased in cells exposed to Nef+ART (Figure 1Q). These indicate that Nef+ART resulted in both decreased APG biogenesis and accelerated APG degradation.

These data suggest an imbalance in astrocyte autophagy with short-term exposure to Nef or ART which is accentuated when Nef and ART are combined.

### 3.2. Nef Alone or in Combination with ART Accelerates APG Maturation in Astrocytes

Our Western blot analysis demonstrated that distinct steps in autophagy were dysregulated by Nef and ART. To validate our Western blot findings, and gain additional information on the changes in the autophagic system, astrocytes were transduced with lentivirus carrying a tandem fluorescent-tagged LC3 (mCherry–GFP–LC3) [70] and then treated with Nef and/or ART, imaged, and puncta quantified as described in Methods. The degree of autophagic flux is reflected by the ratio of yellow and red fluorescent puncta. APG are detected as yellow fluorescent puncta (mCherry^+^GFP^+^) due to colocalization of mCherry and GFP fluorescence, both present in the tagged LC3. When the APG fuses with the lysosome, creating an autolysosome (AL), the GFP fluorescence is quenched by the acidic lysosomal environment, so that AL appear as are red only puncta (mCherry^+^GFP^−^). Under normal conditions, the number of APG (yellow puncta) at any given time is relatively low, since once formed they rapidly mature into AL (red only puncta). In contrast, a block in APG maturation would manifest as increased yellow puncta with decreased red-only puncta.

An advantage of this image-based approach is that it does not require use of chemical degradation inhibitors to estimate flux. However, we included treatments with rapamycin, to induce autophagy, and chloroquine, to inhibit degradation, as controls to show that astrocytes were appropriately responsive to pharmacologic manipulation of flux. As seen in Figure 2A, transduced astrocytes treated with rapamycin had a striking increase in red puncta compared to untreated control, indicating increased APG maturation into AL, as expected in the presence of an autophagy inducer. This is in contrast to chloroquine treatment, which demonstrated only yellow puncta (Figure 2A), as well as an apparent increase in the total number of puncta compared to untreated control. This was also expected because chloroquine dissipates lysosomal pH and prevents GFP quenching such that autophagic vesicles (AV) will accumulate but not degrade. Untransduced cells displayed no puncta (Figure 2A). Untreated astrocytes had about 30% APG and 70% AL (Figure 2A,C), reflecting their basal level of fully functional autophagic flux.

Astrocytes treated with Nef (Figure 2A) displayed a 37% lower mean number of APG per cell, and a 108% increase in AL, relative to control, respectively (Figure 2B). The decrease in APG was a result of faster maturation into AL, as shown by analyses of the percent of APG and AL that comprise the total AV. Nef significantly decreased the percent APG relative to control (23% compared to 32%, Figure 2C; *p* < 0.005), and increased percent AL (74% compared to control 66%, Figure 2C; (*p* < 0.05), reflecting an accelerated maturation of APG toward AL after Nef. These data show that Nef increased APG maturation to AL but did not induce APG biogenesis, agreeing with the Western blot data.

Treatment with ART caused a mean 20% reduction in total autophagic vesicles compared to control cells (Figure 2A,B). This reduction was due to a decrease in both APG and AL relative to control (42% and 7%, respectively, Figure 2B). Additionally, the percent APG comprising total AV was 7% lower than control (Figure 2C), although not significant. These indicate decreased total AV, confirming an inhibitory effect of ART on autophagy at the level of induction/ biogenesis, with less effects on maturation, which are both supportive of our Western blot data.

Concomitant treatment with Nef and ART reduced the number of total AV and accelerated maturation of APG into AL. There was an 11% decrease in total AV after Nef+ART treatment relative to control (Figure 2A,B), with APG comprising 24% and AL comprising 74% of total AV (Figure 2C). This is significantly decreased and increased, compared to control with 32.4% and 66.6%, respectively (Figure 2C; *p* < 0.005 and *p* < 0.05). These data indicate that Nef+ART decreased autophagy induction/APG biogenesis and accelerated maturation of already formed APG into AL, supporting the dual effects seen by Western blotting.

### 3.3. Nef and/or ART Decrease the Number of APG in Human Astrocytes

**Figure 3:**
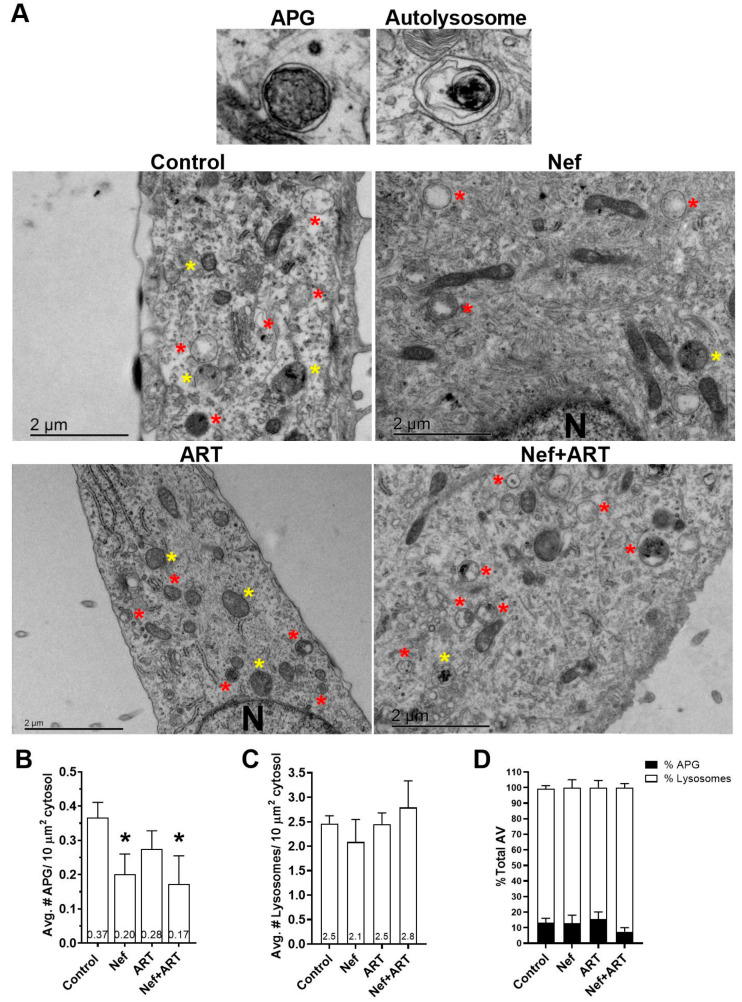
Ultrastructural analysis of APG and lysosomes after treatment with Nef and/or ART. Primary human astrocytes were treated with extracellular Nef and/or ART for 24 h and prepared for and analyzed by transmission electron microscopy as described in Methods. (**A**) Representative APG and autolysosome, and images of control, Nef, ART, and Nef+ART treated cells. Yellow stars = APG; red stars = Lysosomes; N = nucleus. (**B**) The mean number of APG per 10 μm^2^ cytosol. (**C**) The mean number of lysosomes per 10 μm^2^ cytosol. (**D**) Mean percent APG and lysosomes of total AV in control, Nef, ART, and Nef+ART treated astrocytes. Error bars represent SEM. *n* = 2 independent experiments, with a total of 23–27 cells per condition analyzed. *, *p* < 0.05 by Mann-Whitney test.

To obtain further information on possible effects of Nef and/or ART on the autophagic/lysosomal system, we used transmission electron microscopy and morphometric analyses on primary astrocytes treated for 24 h with Nef and/or ART. We did not use an inhibitor of lysosomal degradation to halt the dynamic autophagic process, in order to capture a snapshot of the steady-state of autophagy compartments after treatments. We scored images for numbers of APG as well as for numbers of lysosomes. Figure 3A provides an example APG and an autolysosome, identified by the presence of degrading cytosolic material, and representative images from control and Nef and/or ART treated cells. Analyses showed that treatment with Nef and Nef+ART for 24 h caused a significant reduction in the mean number of APG per 10µm^2^ cytosol (46% and 54%, respectively, Figure 3A,B; *p* < 0.05). ART also decreased the mean number of APG per 10µm^2^ cytosol to 24% of control (Figure 3B), although not significantly. The numbers of lysosomes are not significantly different than control (Figure 3C), but it is important to note that all types of lysosomes were included in the analysis, since AL resulting from autophagy cannot be differentiated from other subtypes of lysosomes on the basis of morphological criteria alone. There was a 44% decrease in percent APG, and 107% increase in lysosomes after Nef+ART treatment (Figure 3D). This snapshot is consistent with our biochemical analyses, confirming the reduction in APG and accelerated maturation of APG into AL after treatments.

### 3.4. Nef and/or ART induce “Long-Term” Autophagy Imbalance in Astrocytes

**Figure 4:**
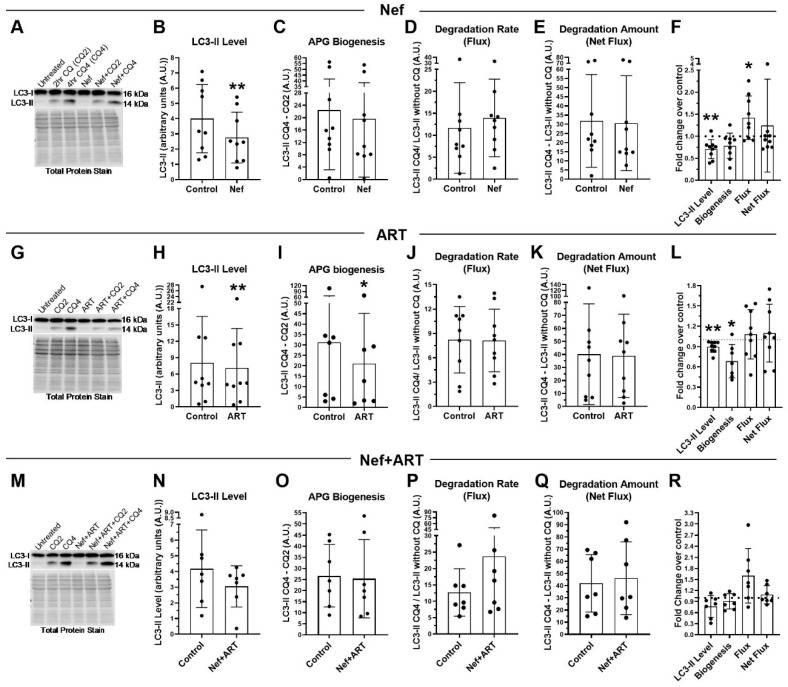
LC3-II steady-state level, APG biogenesis and APG degradation after treatment with Nef and/or ART for 7 days. Primary human astrocytes were treated daily for 7 days with extracellular Nef and/or ART, without and with chloroquine (CQ) for the last 2 or 4 h, then LC3 Western blotting was performed. Steady-state level, biogenesis, flux and net flux for LC3-II were calculated as described in Methods. **(A**,**G**,**M**) Representative LC3-II Western blots after A. Nef, G. ART, or M. Nef+ART treatment. B-E, H-K, and N-Q show individual experiments (dots) and the means of the experiments (bars). (**B**,**H**,**N**) LC3-II steady-state level in control and B. Nef, H. ART, or N. Nef+ART treated astrocytes. (**C**,**I**,**O**) APG biogenesis in control and C. Nef, I. ART, or O. Nef+ART treated cells. (**D**,**J**,**P**) Rate of APG degradation (Flux) in control and D. Nef, J. ART, P. Nef+ART treated astrocytes. (**E**,**K**,**Q**) Amount of APG degradation (net flux) in control and E. Nef, K. ART, or Q. Nef+ART treated cells. (**F**,**L**,**R**) show the fold changes of the same autophagy parameters after treatment with F. Nef, L. ART, and R. Nef+ART over controls of individual experiments (dots) and the means of the fold changes of the experiments (bars). Controls were set to 1, represented by the dashed lines. Error bars depict SD. *n* = 7–11; *, *p* < 0.05; **, *p* < 0.005 by the Wilcoxon Matched-Pairs Signed Rank test or the Wilcoxon Signed Rank test.

PLWH have persistent exposure to Nef due to its production from viral reservoirs, despite ART, and when undergoing treatment, have continued exposure to antiretroviral drugs by dosing necessity. To approximate more closely the experience of PLWH and examine astrocyte autophagy dynamics on a longer-term basis, we chose to extend treatment duration with Nef and/or ART to 7 days, and again analyzed LC3-II by Western blotting. After 7 days of daily Nef treatment, LC3-II steady-state level remained significantly decreased, 31% less than control (Figure 4A,B, or 0.29-fold less; *p* < 0.005). Degradation rate (flux) remained 120% accelerated relative to control (Figure 4A,D), or 1.4-fold above control (Figure 4A,F; *p* < 0.05). At this extended time point, there was not a significant change from control in APG biogenesis after Nef treatment (mean 19.7 compared to control mean 22.4, Figure 4A,C), nor in the amount of APG degraded (net flux) (mean 35.7 compared to control mean 31.8, Figure 4A,E), confirming that accelerated degradation, and not reduced biogenesis, leads to the significantly reduced LC3-II level.

After 7 days of daily ART, LC3-II steady-state level remained significantly 11% less relative to control (Figure 4G,H; *p* < 0.005), or 0.11-fold less than control (Figure 4L; *p* < 0.005). This is again resulting from a significant decrease in APG biogenesis (33% less than control, Figure 4G,I; *p* < 0.05; or 0.32-fold less, Figure 4L; *p* < 0.005). Similar to the 24 h treatment, there were different groups of response to 7 days of ART (Figure 4K,L). At this extended time point, there are three groups present: one with increased degradation (mean 1.49-fold increase; *n* = 3, Figure 4L), one with no change in degradation relative to control (mean 1.1-fold; *n* = 2, Figure 4L), and one with decreased degradation relative to control (mean 0.62-fold decrease; *n* = 2, Figure 4L). The mean fold increase in the high responders is not as high as the mean fold increase in the high responders after 24 h of treatment (Figure S2). The third group with decreased flux was not detected after 24 h of treatment (Figure 1D). These data suggest that prolonged ART exposure may lead to reduced overall autophagic flux, with decreased biogenesis and degradation.

LC3-II steady-state level remained decreased after treatment with Nef+ART for 7 days, at 27% less than control (Figure 4M,N), or 0.24-fold less than control (Figure 4M,R), although this was not significant. The decreased biogenesis and increased degradation rate (flux) present after 24 h of treatment with Nef+ART was maintained after 7 days. Biogenesis was 10% less than control (Figure 4M,O,R), and degradation was accelerated to 187% above control (Figure 4P), or 1.6-fold above control (Figure 4R). These data are consistent with the changes noted after 24 h of treatment (Figure 1M–R).

We do not have statistical power to compare 24 h results to 7 days results. However, we include graphs for comparison of autophagy parameters at the two time points (Figure S2). These data demonstrate that changes present at 24 h after Nef and/or ART remain present after 7 days of daily treatment though the changes after 7 days is not as dramatic after 24 h.

### 3.5. Nef and/or ART Compromise p62-Mediated Selective Autophagy Short and “Long Term”

Autophagosomes can engulf cytosolic components that are near to the forming autophagosome in a non-selective manner (“in-bulk”) [40]. Autophagy can also be selective through autophagy adaptors and/or receptors that bring specific cargo to the forming autophagosome for selective lysosomal degradation [40,74]. P62 is a well-characterized autophagy receptor that brings ubiquitinated cargo, among other cargo types, to the autophagosome for selective degradation by autophagy [74]. P62 is trapped with cargo within the APG as the APG membrane closes and, consequently, degraded in the autolysosome along with the cargo. Therefore, whereas measurement of LC3-II degradation provides information on overall autophagy independent of its selectivity, lysosomal degradation of p62 can be used as a surrogate to infer changes in selective autophagy [68]. To determine whether Nef and/or ART interfere with selective autophagy, or impact only non-selective autophagy as we showed by LC3-II analysis, we performed Western blot analysis of p62 after 24 h and 7 days of daily Nef and/or ART treatment as described in Methods. We analyzed p62 turnover in a similar way as we assessed LC3-II turnover to measure autophagy flux. We show data resulting from normalization to total protein. There was no significant difference between p62 normalized to total protein relative to p62 normalized to the 25 kDa band (*p* = 0.62 by paired T-test; data not shown).

**Figure 5:**
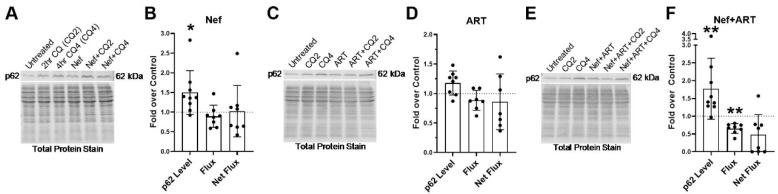
p62 lysosomal degradation after treatment with Nef and/or ART for 24 h. Primary human astrocytes were treated with extracellular Nef and/or ART for 24 h, without and with chloroquine (CQ) for the last 2 or 4 h, after which p62 Western blotting was performed. Steady-state level, flux and net flux for p62 were calculated as described in Methods. (**A**,**C**,**E**) Representative p62 Western blots after A. Nef, C. ART, or E. Nef+ART treatment. (**B**,**D**,**F**) show the fold changes of autophagy parameters after B. Nef, D. ART, and F. Nef+ART treatment over control of individual experiments (dots) and the means of the fold changes of the experiments (bars). Controls were set to 1, represented by the dashed lines. Error bars depict SD. *n* = 7–9; *, *p* < 0.05; **, *p* < 0.005 by the Wilcoxon Signed Rank test.

After 24 h of Nef treatment, there is significantly increased p62, 1.5-fold over control (Figure 5A,B; *p* < 0.05). This increase is likely due to a 12% decrease in the degradation rate (flux) of p62 after Nef treatment (Figure 5B). ART also increases p62 1.2-fold above control after 24 h of treatment. This is also due to a 12% decrease in the rate of degradation (flux) as well as a 15% decrease in the amount of p62 degradation (net flux), though these are not significant (Figure 5C,E). Treatment with Nef+ART for 24 h significantly increased p62 1.8-fold over control (Figure 5E,F; *p* < 0.005). This increase is due to a significant 35% decrease in p62 degradation rate (flux) (Figure 5F; *p* < 0.05) and a 52% decrease in degradation amount (net flux) (Figure 5F). These data indicate that Nef and/or ART can impair p62 lysosomal degradation in astrocytes after 24 h of treatment. It is likely that the reduced formation of APG after Nef and/or ART treatment results in the high levels of p62 in these cells.

**Figure 6:**
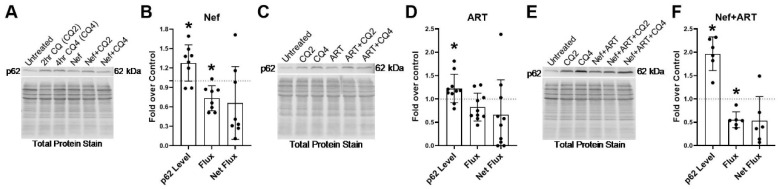
p62 lysosomal degradation after treatment with Nef and/or ART for 7 days. Primary human astrocytes were treated with extracellular Nef and/or ART daily for 7 days, without and with chloroquine (CQ) for the last 2 or 4 h, after which p62 Western blotting was performed. Steady-state level, flux and net flux for p62 were calculated as described in Methods. (**A**,**C**,**E**) Representative p62 Western blots after A. Nef, C. ART, or E. Nef+ART treatment. (**B**,**D**,**F**) show the fold changes of autophagy parameters after B. Nef, D. ART, and F. Nef+ART treatment over control of individual experiments (dots) and the means of the fold changes of the experiments (bars). Controls were set to 1, represented by the dashed lines. Error bars depict SD. *n* = 6–10; *, *p* < 0.05; by the Wilcoxon Signed Rank test.

To evaluate potential changes in selective autophagy, through analysis of p62 lysosomal degradation, on a longer-term basis, we extended treatment duration with Nef and/or ART to 7 days, and analyzed p62 by Western blotting. All three treatments led to a significant increase in p62 steady-state level relative to control (Figure 6). After Nef treatment, there was a significant 1.3-fold increase (Figure 6A,B, *p* < 0.05). There was a significant 1.2-fold increase in p62 steady-state level after 7 days of ART treatment (Figure 6C,D; *p* < 0.05). Nef and ART combined led to a significant 2-fold increase in p62 steady-state level above control (Figure 6E,F; *p* < 0.05). This increase was due to a decrease in both the rate of p62 degradation (flux) and amount of p62 degradation (net flux). Nef significantly decreased flux 27% (*p* < 0.05) and net flux 35% (Figure 6B) relative to control. ART also decreased flux 17% and net flux 35% relative to control, though these were not significant (Figure 6D). Nef+ART significantly decreased flux 45% (*p* < 0.05) and net flux by 47% (Figure 6F). These data demonstrate that Nef and/or ART impair selective autophagy of p62 both short term and “long term.”

**Figure 7:**
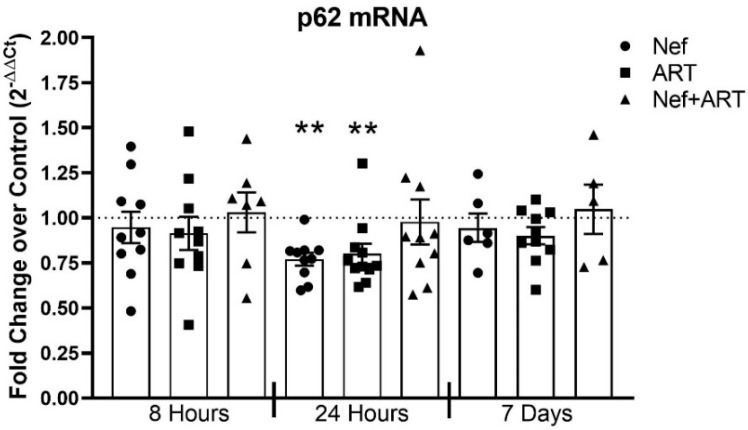
p62 mRNA transcription after treatment with Nef and/or ART for 8 or 24 h or 7 days. Primary human astrocytes were treated with extracellular Nef and/or ART for 8 or 24 h or daily for 7 days, and qRT-PCR was performed. Graph depicts the fold change of p62 mRNA over control of individual experiments (dots), and the mean of all experiments (bars). Control is depicted as the dashed line at 1. Error bars represent SEM. *n* = 5–10; **, *p* < 0.005; by the Wilcoxon Signed Rank test.

To confirm further that the increased amount of p62 protein after Nef and/or ART treatment was due to decreased p62 degradation rather than to upregulation in p62 transcription, we performed qRT-PCR after 8 or 24 h or 7 days of Nef and/or ART treatment. P62 transcription is not changed relative to control after 8 h of treatment, nor after 7 days of daily treatment (Figure 7). After 24 h of Nef or ART treatment, there is a significant decrease in p62 mRNA relative to control, 23% and 25%, respectively (Figure 7; *p* < 0.05). While after 24 h of Nef+ART treatment there is no overall difference compared to control, 7 out of the 10 experiments show decreased mRNA after Nef+ART relative to control (Figure 7). No change in p62 mRNA after treatments relative to control, and even decreased p62 mRNA after treatments relative to control, supports our conclusion that Nef and/or ART increase p62 level because lysosomal p62 degradation is inhibited. Thus, Nef and/or ART impair p62 selective autophagy.

We do not have statistical power to compare 24 h p62 results to 7 days p62 findings. However, we include graphs for comparison of p62 changes at the two time points (Figure S3). These data demonstrate that changes present at 24 h after Nef and/or ART remain present after 7 days of daily treatment.

**Figure 8:**
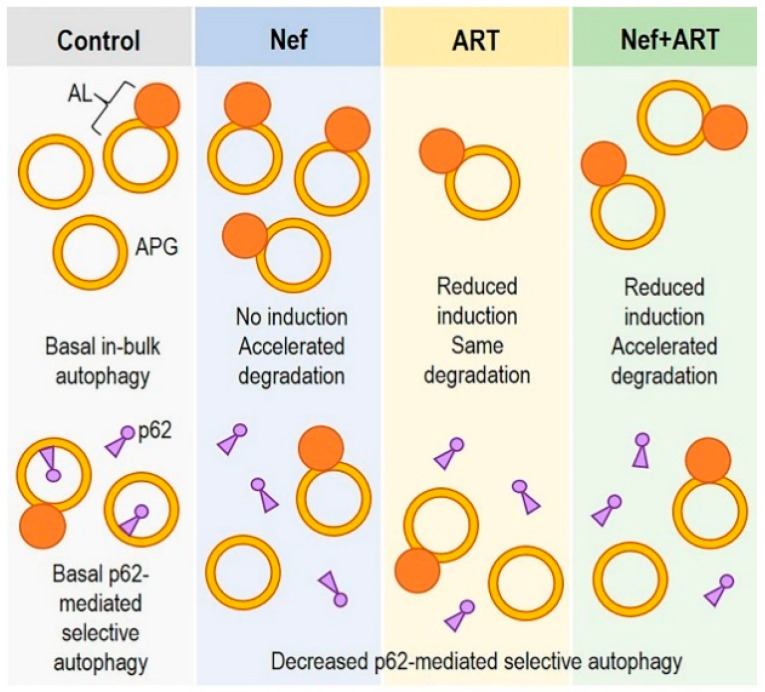
Schematic depicting in-bulk autophagy dysregulation and inhibition of p62-medicated selective autophagy in astrocytes after Nef and/or ART treatment.

In summary, our findings show that extracellular Nef and/or ART induce autophagy imbalance in astrocytes and may differently interfere with “in-bulk” and “selective” autophagy (Figure 8). Over long periods of exposure to extracellular Nef and/or ART, this may result in astrocyte dysfunction and loss of homeostasis, leading to neuronal dysfunction and development of HAND.

## 4. Discussion

Autophagy is essential for maintaining CNS cell homeostasis, especially in long-lived cells such as astrocytes. Autophagy not only clears damaged organelles and proteins, and protects against stress and starvation, but is also linked to other vital cell processes such as glutamate uptake, response to ROS, cytokine regulation, and to apoptosis [75,76,77,78,79,80]. Autophagy dysregulation has been identified in many neurodegenerative diseases, and may also contribute to HAND pathogenesis [28,38,54,55,56,57,58,59,60,61,62,63,64]. Mechanisms underlying development of HAND, particularly in the current ART era, however, remain minimally defined. This is the first report, to our knowledge, to describe an imbalance in autophagy in astrocytes resulting from exposure to extracellular Nef, as well as to commonly prescribed antiretroviral drugs in a concentration consistent with CSF levels. We find that Nef accelerated APG degradation, ART decreased APG biogenesis, and exposure to concomitant Nef and ART resulted in both decreased APG biogenesis and accelerated APG degradation. These effects manifested with short-term treatment, confirmed with three independent and complimentary techniques, and were maintained after “long-term” exposure. We also find that selective autophagy, quantified by p62 lysosomal flux, may be inhibited by all three treatments after both short- and “long-term” treatment. We propose that this imbalance in autophagy will disrupt astrocyte homeostasis and function, and thus has important implications for PLWH and their development of HIV-associated neurocognitive disorders. Our data also indicate some potential interventional strategies to mitigate this burdensome comorbidity.

Upregulation of autophagic flux is a normal cellular response to stress and is usually manifested by increased APG biogenesis coupled with increased APG degradation. While we found accelerated APG degradation after Nef, there was not an accompanying increase in APG biogenesis, as would be expected if autophagic flux were induced as a normal response to the presence of a flux inducing stimuli. Similarly, degradation after ART treatment was variably impacted while APG biogenesis was consistently decreased, and astrocytes treated with concomitant Nef and ART had defects in both of these steps in autophagy. The changes induced by Nef and/or ART in biogenesis and degradation are not balanced by appropriate changes in the other steps of autophagic flux, suggesting that the alterations did not result from the normal cellular response to stress. This indicates that Nef and ART cause abnormalities in distinct steps of autophagy, thus creating an imbalance in flux. These effects were not due to the inclusion of chloroquine. First, our experiments with the dual-fluorescent LC3 reporter corroborate our Western blot data, and chloroquine was not used to assess flux in the reporter experiments. Second, we found similar changes in synthesis and degradation after treatment with Nef and/or ART when ammonium chloride plus leupeptin were used to inhibit lysosomal degradation instead of chloroquine (data not shown). Additionally, treatment with chloroquine lasted 4 h. This short duration is unlikely to cause any significant toxicity to the astrocytes. Thus, these effects on autophagy are due specifically to Nef and/or ART.

Autophagy is upregulated by stimuli typically within a few to several hours. We performed time course experiments in which we evaluated flux after 6 and 11 h of Nef and/or ART treatment but did not see significant changes relative to control cells (data not shown). We found that APG biogenesis and degradation were most altered after 24 h of treatment, long after the expected duration of autophagic upregulation. Moreover, the changes to autophagy induced by Nef and/or ART persisted over 7 days of treatment, suggesting there are long-lasting effects of these treatments. We postulate that ongoing abnormalities induced by Nef and/or ART ultimately contribute to long-term imbalance in autophagic flux, leading to autophagy exhaustion and failure. Similar defects in autophagy have been shown in neurodegeneration as well as in physiological aging [81,82,83]. To this point, it has also been postulated that autophagy dysregulation in CNS cells from people with HIV encephalitis, a type of HAND, reflects a picture of autophagy exhaustion and accelerated aging [55]. This hypothesis is supported by our Western blot data in that the fold changes in LC3-II level, biogenesis, and degradation relative to control were not as pronounced after 7 days as compared to 24 h, suggesting that the cells could not keep up with the ongoing imbalance between biogenesis and degradation induced by the treatments. Autophagy is primarily regulated by post-translational modifications and changes in the subcellular location of the proteins comprising the autophagic machinery. While there is increasing appreciation for the role of transcription in regulating autophagy dynamics, it is still believed to be a secondary regulatory mechanism. In support of our exhaustion hypothesis, we have preliminary data (not shown) that transcription of select autophagy-related genes is increased after 7 days of treatments, but not at 24 h, suggesting an upregulation in gene expression to compensate for the dysregulation at the protein level resulting from prolonged treatment.

Our data demonstrate that ART decreased APG biogenesis in astrocytes, suggesting inhibited autophagy initiation and/or APG elongation. Another study found autophagy inhibition at the level of APG biogenesis in brain endothelial cells after exposure to efavirenz, a non-nucleoside reverse transcriptase inhibitor. Efavirenz inhibits the activity of the Beclin-1/Atg14/PI3KIII complex, leading to decreased PI3P [60], which is needed for the recruitment of downstream proteins responsible for APG membrane nucleation. Autophagy initiation can also be inhibited through the bcl-2–beclin-1 interaction [84]. There are several publications demonstrating changes in bcl-2 in the presence of ART, either in PBMC from PLWH or in cancer cell models, but the effects of the antiretrovirals are varied [85,86,87,88,89,90,91,92], suggesting a drug-, cell- and/or disease process type-specific effect of antiretrovirals on bcl-2. To our knowledge, there are no studies linking antiretroviral drugs to autophagy changes through changes in bcl-2. In a Parkinson’s disease model, APG formation was inhibited due to ATG9 mislocalization, resulting in toxic α-synuclein accumulation [93]. ATG9 coordinates membrane transport from the donor organelle to support elongation of the double-membraned APG. An initiation defect is also linked to ALS/frontotemporal Dementia due to inhibited trafficking of ULK1 to autophagy initiation sites [94]. The ULK1 complex translocates to autophagy initiation sites, regulating recruitment of downstream signaling and effector molecules that ultimately lead to the formation and expansion of the APG membrane. Ultimately, fewer APG results in insufficient amount of cargo degradation, leading to accumulation of damaged organelles, mutant or misfolded proteins, and toxic macromolecule aggregates. It will be important to understand the mechanism by which ART inhibits APG biogenesis in astrocytes, particularly as long-term ART exposure, such as in those undergoing treatment for HIV, may lead to accumulation of cargo intended for degradation, causing toxicity and disruption to astrocyte homeostasis, ultimately contributing to CNS damage and HAND.

We also show that Nef and/or ART may inhibit p62-mediated selective autophagy. In contrast to the accelerated LC3-II clearance upon Nef or Nef+ART exposure, p62 flux was consistently reduced after treatments. These results suggest that only APG involved in “in-bulk” autophagy, and not APG containing p62-tethered cargo, underwent accelerated degradation. Further investigation is needed to determine whether differences in the sequestered cargo result in slower APG maturation, or if specific cargo intended for degradation through autophagy may actually be excluded from degradation in the presence of Nef and/or ART. The exclusion of particular cargo, for example, ubiquitinated protein aggregates which is one type of cargo selectively targeted by p62 into APG, will negatively impact astrocyte homeostasis leading to functional decline, loss of viability, and neurodegeneration. Accumulation of ubiquitinated proteins as a result of inhibited autophagy has been described in Huntington’s disease and in ALS/frontotemporal dementia [95,96,97]. Selective autophagy malfunction contributes to the pathogenesis of common neurodegenerative diseases (reviewed in [98]), and there is a prominent role for astrocytes in these conditions [99]. We have preliminary data indicating that at least poly-ubiquitinated proteins are accumulating after 7 days of treatment with Nef and/or ART (data not shown). It is attractive to propose that the inhibition of autophagy upon Nef and/or ART exposure described in this work contributes to HAND pathogenesis by inducing neuropathogenic protein accumulation. This is a focus of our future studies.

P62 links autophagy to one of the major cellular defense mechanisms against oxidative stress, the NRF2-Keap1 system [100]. In homeostasis, the cytosolic transcription factor nuclear factor erythroid 2-related factor 2 (Nrf2) is bound to kelch-like ECH-associated protein 1 (Keap1), an adaptor of the cullin-3-type ubiquitin ligase complex for Nrf2. This results in constitutive degradation of Nrf2 by the ubiquitin–proteasome system. However, with exposure to reactive oxygen species and nitric oxide, modifications to the Keap1 protein result in Nrf2 stabilization and translocation into the nucleus where transcription of numerous cytoprotective genes is induced. p62 competes with Nrf2 for Nrf2′s binding site on Keap1 [101]. Thus, excess p62, from perturbations in selective autophagy, will cause stabilization of Nrf2 with subsequent transcription of Nrf2 target genes [101]. The promotor for p62 contains an antioxidant response element (ARE) to which Nrf2 will bind. Thus, activation of the Nrf2 system by p62 establishes a positive feedback loop [102]. Persistence of p62 in the astrocyte cytosol resulting from Nef- and/or ART-induced autophagy inhibition could contribute further to cellular proteotoxicity through its well characterized role in aggregosome formation and NRF2-mediated signaling. While we show that changes in p62 levels were a direct result of reduced autophagic degradation and not from transcriptional changes, we also cannot exclude induction of the Nrf2 system with increased transcription of other Nrf2 targets. This remains an important area to study.

One advantage of working with primary human astrocytes is that they enable identification of individual differences in the autophagy response to ART treatment. We found interesting groupings of changes in APG degradation rate (flux) after both 24 h and 7 days of ART treatment. There was one group of independent experiments showing increased degradation, one with no change, and one group showing decreased degradation, relative to control after the longer treatment duration. These differing results may be due to the inherent variability among primary cells. This variability may reflect individuals’ experiencing different types and severity of side effects when taking medication. Antiretroviral drugs may variably impact autophagy degradation in different individuals. Coupled with a consistent decrease in APG biogenesis, this varying impact on degradation highlights that each step of autophagy may be impacted differently by ART. A recent publication showed that LC3-II was slightly increased in primary human astrocytes after treatment with an antiretroviral cocktail of emtricitabine, ritonavir, and atazanavir, yet the combination of lopinavir, abacavir and raltegravir slightly decreased LC3-II after treatment of the same cells [38]. We did not test the effects of individual drugs on autophagy because PLWH take a cocktail of antiretroviral drugs, but this would be of interest to examine. Differential effects on biogenesis as compared to degradation are likely mediated by distinctive mechanisms, and are potentially different depending on which antiretroviral drug, individually or in combination with other drugs, is being studied.

Our findings with ART treatment also have important implications for people taking pre-exposure prophylaxis (PrEP) long term to prevent HIV infection, which is currently comprised of tenofovir and emtricitabine, two of the three antiretroviral drugs we studied. This is particularly noteworthy as the number of individuals taking PrEP increases. Long-term neurologic effects of PrEP have yet to be determined, but the effects of antiretrovirals on autophagy in CNS cells are, and should be, an important consideration during new antiretroviral drug development, not for only for those infected with HIV at risk for developing neurocognitive dysfunction, but for those actively trying to prevent HIV infection with PrEP.

We found that extracellular HIV Nef increased APG maturation in astrocytes. We chose to use exogenous Nef because while a small percentage of astrocytes are believed to be infected with HIV [15,16,17,18,19,20,21], a majority of astrocytes will be exposed to extracellular Nef produced by surrounding infected cells. In this way, we were able to determine the effects of extracellular Nef on astrocyte autophagy. Our findings are in contrast to one published report describing decreased APG degradation in human astrocytes overexpressing Nef [62]. This suggests that the effects of extracellular Nef may be different from the effects of Nef when expressed within astrocytes by HIV infection or by in vitro transfection. Extracellular Nef is thought to recapitulate the same activities described for cells that express Nef as a result of HIV infection or transfection with Nef-expressing vectors [26]. The majority of these Nef activities were described in model systems where the mechanism of Nef entry into the bystander cell was by tunneling nanotubes or by uptake of exosomes containing Nef, implying that delivery of Nef through these means enables Nef to access the same cell compartments that Nef accesses when expressed from within the infected/transfected cell. Transfer of Nef by nanotubes or exosomes is unlikely in our model system as we did not genetically overexpress Nef within the astrocytes. It is possible that Nef was able to enter the cells by an unknown mechanism. Exogenous Nef has been shown to accumulate in immature dendritic cells, and IgD+ B cells by a mechanism not yet defined [31,32]. The mechanism of Nef’s entry into a bystander cell may dictate what cellular compartment Nef can access. Thus, the effects that Nef can mediate inside a bystander cell may be different depending on the mechanism of Nef entry into a cell. This may explain why we found accelerated APG degradation after exposure to extracellular Nef, rather than decreased APG degradation. Alternatively, the accelerated APG degradation may be due to actions of extracellular Nef on astrocyte surface receptors, rather than by mediating its effects from within the intracellular environment. Nef was shown to bind CXCR4, leading to apoptosis of CD4+ T cells [30]. This underscores the important contribution of viral protein–cell surface receptor interactions to changes in intracellular processes, including autophagy, which is linked to many other cell functions. It is therefore of interest to define clearly the effects of extracellular or intracellular Nef on astrocytes to appreciate fully how the manipulation of autophagy by Nef contributes to HAND pathogenesis.

Increased APG maturation is often accompanied by increased lysosomal degradation capacity, which is mediated by the master transcription factor EB (TFEB). TFEB regulates the Coordinated Lysosomal Expression and Regulation (CLEAR) network that, in turn, regulates multiple cell processes including autophagy, lysosomal biogenesis, and lysosomal secretion [103]. In HIV-infected macrophages, Nef has been shown to inhibit autophagy, at least in part, through changes in the cellular location and activity of TFEB [51]. In contrast, we found that exposure to extracellular Nef accelerates APG clearance in astrocytes. It is possible that Nef is acting selectively in one part of the CLEAR network, and this may be cell-type specific, or an effect of extracellular Nef that is different from intracellular Nef, as discussed. Determining whether the effect of Nef on astrocyte autophagy is independent of TFEB and the impact that Nef and/or ART have on TFEB-mediated signaling require additional examination.

Treating astrocytes with both Nef and ART was to model the experience of PLWH on ART, with ongoing Nef exposure even in the presence of antiretroviral drugs. That ART did not mitigate the changes in autophagy induced by Nef is not surprising given that the mechanisms of action of the drugs in the cocktail we used inhibit viral replication rather than targeting Nef specifically. Currently, there are no therapies to target Nef. As shown here, the off-target effects of antiretroviral drugs have the potential to modulate autophagy, and not necessarily in a beneficial manner.

The mechanisms underlying the changes in both in-bulk and p62-mediated selective autophagy identified here caused by Nef and ART remain unknown and are important for further study. Identifying Nef- and ART-induced changes in signal transduction and transcription programs, binding partners, and details regarding autophagosome cargo would increase our understanding of the role of astrocytes in HAND pathogenesis, and identify potential therapeutic targets for HAND treatment. To modulate autophagy to mitigate HAND is a very attractive idea. Autophagy manipulation is the focus of drug development in multiple disciplines, including in the neurodegeneration field. One cautionary note, however, is that developing novel drugs to modulate autophagy to produce beneficial effects without causing negative effects on bystander cells will be a formidable task. Additionally, we do not yet know how the Nef- and ART-induced imbalance in autophagy impacts astrocyte function with regard to regulating the neuronal microenvironment. Autophagy and its associated proteins are directly and indirectly linked to the regulation of glutamate uptake, the response to reactive oxygen species, cytokine production, amino acid metabolism and apoptosis [76,77,78,79,80]. It is possible that the imbalance in autophagy impacts one or all of these processes, thus disrupting homeostasis, losing the ability to support neurons. Understanding the importance of autophagy to other astrocyte processes would expand our knowledge of not just HAND pathogenesis, but also of other neurodegenerative disorders. Studies in this arena would also identify potential therapeutic targets to mitigate HAND, and possibly other neurocognitive diseases.

## Supplementary Materials

The following are available online at https://www.mdpi.com/2073-4409/9/6/1426/s1, Figure S1. Representative LC3-II Western blot with corresponding total protein stain for comparison of the two normalization approaches for LC3-II. Figure S2: Comparison of APG biogenesis and degradation fold changes after 24 h or 7 day treatment with Nef and/or ART. Figure S3. Comparison of p62-II level, degradation rate, and degradation amount after 24 h or 7 days treatment with Nef and/or ART.
