# Supplementary material for: HIV Nef and Antiretroviral Therapy Have an Inhibitory Effect on Autophagy in Human Astrocytes that May Contribute to HIV-Associated Neurocognitive Disorders
Source: Cells. 2020 Jun 9;9(6):1426. doi: 10.3390/cells9061426 (PMC7349791; doi:10.3390/cells9061426)
Supplement: Supplementary file 1 [file cells-09-01426-s001.pdf]

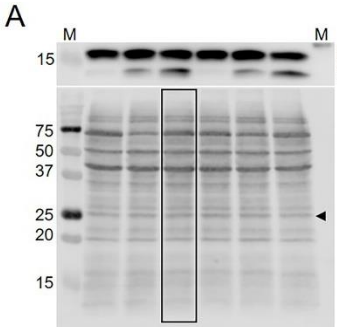

B

	Normalized LC3-II					
Total Protein	1.5	8.9	22	1.2	7.8	16
25 kDa Band	2	8.8	22	1.2	7.3	16

Figure S1. Representative LC3-II Western blot with corresponding total protein stain for comparison of the two normalization approaches for LC3-II. A. LC3-II was normalized either to total protein, represented by the box, or to a 25 kDa band, represented by the arrowhead, as described in Methods. M= molecular weight marker; marker values displayed to the left of the large blot. B. The normalized LC3-II numbers obtained from the Western blot shown in panel A, after normalization to total protein (top) and after normalization to the 25 kDa band (bottom). There was no significant difference between the corresponding normalized LC3-II numbers (p=0.15, by paired T-test).

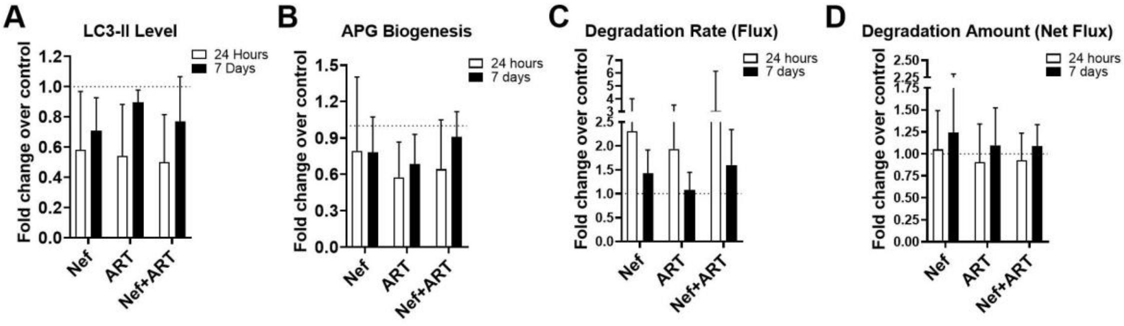

Figure S2. Comparison of LC3-II steady-state level, APG biogenesis, degradation rate and degradation amount after 24h or 7 day treatment with Nef and/or ART. A-D. Mean fold changes of A. LC3-II level, B. APG biogenesis, C. Rate of APG degradation (flux), and D. Amount of APG degradation (net flux) after Nef and/or ART treatment over control. Control is represented by the dashed lines at 1. *n* = 7 - 11. Error bars depict SD.

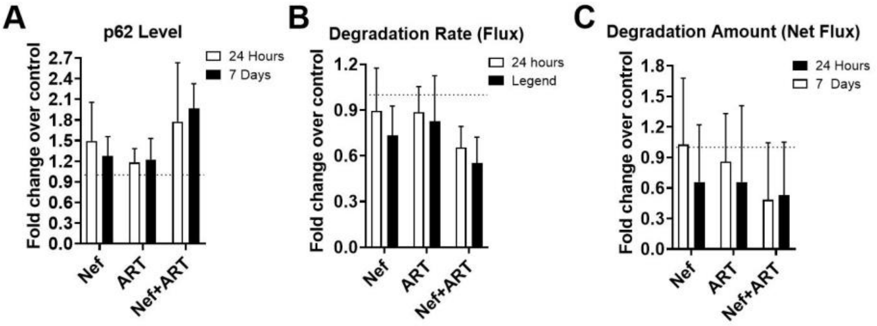

Figure S3. Comparison of p62-II Level, degradation rate, and degradation amount after 24h or 7 day treatment with Nef and/or ART. A-C. Mean fold changes of A. p62 level, B. Rate of p62 degradation (flux), and C. Amount of p62 degradation (net flux) after Nef and/or ART treatment over control. Control is represented by the dashed lines at 1. *n* = 5 - 10. Error bars depict SD.
